# Non-cohabiting Partners’ Economic Characteristics and the Transition to Living Together in Germany: A Couple-Level Perspective

**DOI:** 10.1007/s10680-025-09740-y

**Published:** 2025-10-08

**Authors:** Valeria Ferraretto, Nicole Hiekel, Agnese Vitali

**Affiliations:** 1 Department of Sociology and Social Research, University of Trento, Trento, Italy; 2 Max Planck Institute for Demographic Research, Rostock, Germany

**Keywords:** Cohabitation, Employment, Economic characteristics, Gender inequalities, Germany, Union formation

## Abstract

Living together marks an important step in the progression of intimate partner relationships. While existing literature highlights the relevance of both partners’ economic characteristics for union formation, it remains unclear whether both partners’ economic characteristics are equally linked to the transition to co-residence, whether one partner’s traits matter more, and how this varies by gender. We address these gaps by examining how the economic characteristics of women and men in non-residential relationships relate to their transition to co-residence. Using 13 waves (2008–2021) of the German Family Panel pairfam and Cox proportional hazards model, we analyse 7165 non-residential relationships, capturing dyadic data on partners’ employment status, contract type, and income. Our findings show that economic factors significantly influence the likelihood of moving in together. Couples with two employed partners are most likely to move in together, while gender plays a role when only one partner works—this transition being more likely when the man is employed. Among employed individuals, higher income increases the probability of moving in together for both men and women. Additionally, temporary employment raises the likelihood of starting a co-residence exclusively for women. Overall, results suggest that men’s employment status plays a pivotal role in this transition and that union formation is socially stratified among young adults, reinforcing gender inequalities in partnership dynamics.

**Supplementary Information:**

The online version contains supplementary material available at 10.1007/s10680-025-09740-y.

## Introduction

Moving in with a partner represents a significant demographic marker of the transition to adulthood and a crucial transition in the union formation process. In addition to representing a form of institutionalisation of an intimate relationship, co-residence entails the residential relocation of one or both partners (Wagner & Mulder, 2015). However, understanding the conditions under which a couple decides to move in together is complex, as it requires information not only on the characteristics of individuals who have a partner with whom they intend to co-reside but also on the characteristics of the partner. This study examines the role of economic conditions in shaping the transition from living apart to living together among partnered individuals.

Over the past decades, the contexts in which individuals form their unions have changed considerably. In Western countries, various relationship arrangements beyond marriage—such as dating and non-marital cohabitation—are now common. Moreover, some individuals maintain intimate relationships while residing in separate households, a practice known as ‘living apart together’, blurring the once neat distinction between being ‘single’ and ‘partnered’ based on co-residence (Levin, 2004; Sassler, 2010). Non-residential partnerships, defined here as partnerships between individuals who consider themselves to be in an intimate relationship but who do not share a household,^1^ have become increasingly prevalent. According to the latest available estimates, approximately 9% of the adult population in Western Europe was in a non-residential relationship in 2006 (Liefbroer et al., 2015); recent evidence from Spain suggests this proportion has grown (Nishikido & Castro-Martín, 2024). Expanded participation in tertiary education and in the labour market, particularly among women, have influenced attitudes, preferences, and living arrangements, ultimately reshaping patterns of assortative mating. As a result, educational homogamy and hypogamy (i.e., couples in which the woman is as educated or more educated than the man) have become more common than hypergamy (couples in which the man is more educated than the woman) (Van Bavel et al., 2018).

Simultaneously, securing stable, well-paid employment has become increasingly difficult for young adults due to labour market deregulation and economic downturns (Aassve et al., 2013; Mills & Blossfeld, 2005; Sironi, 2018). The prevalence of low-paid, precarious jobs affects not only young adults’ economic prospects but also their desirability as partners and their decisions regarding whether and when to move in with a partner. Unemployment, low income, and temporary employment are associated with lower risks of union formation (Palumbo et al., 2022), delayed marriage (van Wijk et al., 2021; Vignoli et al., 2016), lower fertility (Alderotti et al., 2024; van Wijk et al., 2021, 2022), and increased risks of union dissolution (Di Nallo et al., 2022; Jalovaara, 2013).

Despite extensive research on the economic determinants of union formation, prior studies have largely overlooked a key factor influencing the decision to cohabit: the economic characteristics of non-residential partners. Most existing studies assess the association between individuals’ economic characteristics and their likelihood of forming a co-residential partnership, whether through marriage or non-marital cohabitation, while implicitly assuming that individuals are single before they move in with a partner (Bolano & Vignoli, 2021; Jalovaara, 2012; Kalmijn, 2011; Palumbo et al., 2022; Pelikh et al., 2022). However, the probability of moving in together is naturally higher among individuals who are already in a romantic relationship. Moreover, just as an individual’s own economic characteristics shape their likelihood of cohabiting, their partner’s economic characteristics also play a crucial role (Tables 1, 2).

A handful of studies incorporating information on both partners have demonstrated that the partner’s economic characteristics significantly influence transitions from cohabitation to marriage or separation (Ishizuka, 2018; Jalovaara, 2013; Smock & Manning, 1997). Because of the limited availability of data on couples before they are observed in the same household, no prior research has examined how a partner’s economic characteristics affect the initial transition from a non-residential to a co-residential union. This study fills that gap by adopting a dyadic perspective to identify how partners with different economic resources vary in their likelihood of moving in together. Specifically, we examine whether gendered economic disparities—such as men’s higher earning potential or women’s greater financial precarity—are related to the likelihood and timing of moving in together.

We analyse longitudinal data from the German Family Panel (pairfam) collected annually between 2008 and 2021. These data track individuals and information on their current partner from the start of their intimate relationships, rather than from the start of co-residence, and include detailed information on the occurrence and timing of cohabitation, marriage, and separation. Pairfam provides comprehensive data on the economic characteristics of individuals and their current partner (e.g., level of education, employment status) and additional details on the main respondent (e.g., type of employment contract, income). Germany constitutes an interesting case study given the labour market changes outlined above. Although employment rates for both men and women are relatively high, low wages and temporary jobs are common upon labour market entry (Brady & Biegert, 2017; Goebel et al., 2015). Most young adults in non-cohabiting unions in Germany mention employment-related factors as reasons for not living with their partner (Liefbroer et al., 2015).

This study makes both conceptual and empirical contributions to the literature on the economic determinants of union formation. Conceptually, we advance previous research by adopting a dyadic approach, emphasising how the combined economic characteristics of two partners (rather than one partner only, typically, the respondent in longitudinal studies) influence the transition to co-residence. This perspective allows us to examine (i) the social stratification of entry into cohabitation and (ii) how economic inequalities within couples are associated with entry into cohabitation. Empirically, the use of a large sample of non-cohabiting partnerships reported by individuals born in different cohorts (1971/1973, 1981/1983, 1991/1993, 2001/2003) and the inclusion of higher-order unions allow us to provide a comprehensive view of the economic preconditions for co-residence among contemporary young adults in Germany. By distinguishing the transition to cohabitation from broader partnership formation, our study contributes to a deeper understanding of ongoing change in family formation, gender dynamics, and fertility patterns (Bergström & Moulin, 2022; Esteve et al., 2020; Manning, 2020; Sassler, 2010).

## Background

### Grasping Complexity in the Transition to Co-residential Unions: Insights from Previous Literature

There are many potential reasons for wanting to move in with a partner, ranging from a desire for more privacy and intimacy, to spending more time together, or planning to start a family. However, cohabitation can also be triggered by events such as job changes, housing needs, or a desire to pool financial resources (Sassler & Miller, 2011) and achieve economies of scale (Oppenheimer, 1988). Over the last two decades, the high levels of economic uncertainty associated with globalisation, the Great Recession, and the COVID-19 pandemic has disproportionally affected young adults, worsening their economic conditions more than other age groups (Aassve et al., 2013; Eurofound, 2021; Mills & Blossfeld, 2005; Sironi, 2018) and increasing inequalities in achieving residential independence (Ferraretto et al., 2024; Luppi et al., 2024). During the same period, housing prices have significantly risen across European countries and cities (OECD, 2024). As a result, several studies in Europe (Bolano & Vignoli, 2021; Jalovaara, 2012; Kalmijn, 2011; Palumbo et al., 2022; Pelikh et al., 2022; Vignoli et al., 2016) and in the USA (Bloome & Ang, 2020) have explored the economic determinants of union formation. These studies have looked into factors such as educational attainment, objective and subjective economic uncertainty, income, and parental social class across different countries.

However, mainly due to data limitations, these studies often assumed that individuals without co-residential partners are unpartnered. Thus, the number of people who are considered ‘at risk’ of cohabiting is overestimated, given that only individuals who are in an intimate relationship can move in together. As a result, previous research ignored the selection into non-residential partnerships and, more importantly, studied the transition to co-residence as an outcome of individual rather than couple attributes.

To understand the transition to cohabitation, it is crucial to include information about non-residential partnerships. As such information is collected in the German Family Panel, it has been the empirical basis of a number of studies addressing the determinants of the transition to co-residence; i.e., relationship duration and quality (Ciritel, 2022); geographical distance between partners (Krapf, 2018; Krapf et al., 2022); socio-demographic characteristics; or a mix of these factors (Wagner et al., 2019). Other research has relied on national-level surveys (Meggiolaro, 2010; van der Wiel et al., 2020) or on register data (van der Wiel et al., 2023). In the USA, qualitative and quantitative research has shown that working-class couples tend to move in together faster than their middle-class counterparts (Sassler & Miller, 2011; Sassler et al., 2018).

Non-residential couples differ in their risk of transitioning to co-residence. A number of surveys conducted in various European countries (e.g., Generations and Gender Survey, Spanish Fertility Survey) have identified the following reasons for choosing not to live with a partner: not feeling ready; a desire for independence; practical constraints related to the partners’ financial, housing, or work situations; and the presence of children from previous unions. Findings suggest that most young couples do not live together due to external constraints in the domain of paid work, such as the lack of financial independence, or face challenges related to housing (Castro-Martín et al., 2008; Liefbroer et al., 2015; Nishikido & Castro-Martín, 2024; Régnier-Loilier et al., 2009). Thus, during young adulthood, and especially while still enrolled in education, non-residential unions tend to be temporary arrangements that do not represent an alternative to cohabitation or marriage. Due to increasing rates of union dissolution and repartnering, non-residential unions can also be formed at later stages of the life course; however, these couples are more likely to choose this living arrangement for ideological reasons rather than out of necessity (Liefbroer et al., 2015; Nishikido & Castro-Martín, 2024; Régnier-Loilier et al., 2009). The presence of children from previous relationships also affects partners’ living arrangements, even when the children are adults (de Jong Gierveld & Merz, 2013; van der Wiel et al., 2020). Because of the cross-sectional nature of the surveys discussed here, these studies do not investigate under what conditions non-residential couples are most likely to transition to co-residence.

### The German Context

In Germany, cohabitation has become, over time, the normative way to start a union, with 79% of first unions among people born in the 1970 s starting as non-marital cohabitation (Hiekel, 2014). Marriage has not lost momentum, but it has been steadily postponed and is usually preceded by a period of cohabitation; experiencing more than one cohabitation was relatively rare among the 1970 s cohort but might have become more common among recent cohorts (Hiekel & Fulda, 2018). Given the prevalence of non-marital cohabitation in Germany, our empirical analyses based on moving in together with a partner mainly refer to transitions to non-marital cohabitation.

Young adults usually leave their parents’ home in their early twenties, and leaving home tends to be disconnected from union formation, especially among tertiary educated individuals (Mulder et al., 2002). Patterns of participation in education and in the labour market have been shifting, with women starting to outnumber men in higher education around 2007 (Corti & Scherer, 2021). While the share of women in the labour market is comparatively high, about half of women are employed with part-time contracts; indeed, part-time is the most common schedule for women after their first childbirth. Despite unemployment rates being historically low (around 3% among the working-age population in 2022 according to Eurostat), low-wage work, temporary employment, and income inequality are on the rise in Germany (Brady & Biegert, 2017; Goebel et al., 2015; Kurz et al., 2005). Patterns of employment differ between eastern and western Germany. In eastern Germany, non-marital childbearing and marriage postponement are more common, women remain more attached to the labour market throughout their childbearing years, and the gender pay gap is relatively small (Schäper et al., 2023). In western Germany, the transition to parenthood commonly leads to a massive and prolonged reduction in paid labour among women, and norms around marriage are persistently stronger, with cohabitation being considered a prelude to marriage (Fulda, 2016; Hiekel et al., 2015). Owning a house cannot be considered a precondition for cohabitation in Germany, given the level of approval of long-term renting and the country’s low homeownership rate (47% vs. a European average of 69% in 2022, Eurostat data).

### The Role of Economic Factors in the Transition to Co-residential Unions: Theoretical Perspectives and Hypotheses

In the following section, we draw on existing theories on role incompatibility, uncertainty, and gendered specialisation to explain how the likelihood of transitioning from a non-residential to a co-residential union varies between different types of couple configurations based on their employment status. Next, we draw on similar arguments to formulate hypotheses regarding the differences between employed individuals, addressing the role of contract type and income. These theories are widely used in the literature on the economic determinants of union formation (Ishizuka, 2018; Jalovaara, 2012; Kalmijn, 2011; Palumbo et al., 2022) but they have not been applied to the transition from living apart to living together. In the German context, we assume that experiencing labour market insecurity (because of unemployment or inactivity, temporary employment, or low income) may impede moving in together with a partner. We also expect that women’s and men’s economic characteristics differently affect cohabitation choices.

*Role incompatibility.* There are different reasons why young adults are not active in the labour market. First, being a student is not considered normatively compatible with the role of partner or parent (Blossfeld & Huinink, 1991). Indeed, there is ample evidence that educational enrolment is associated with reduced risks of union formation (Ishizuka, 2018; Jalovaara, 2012; Kalmijn, 2011). A second reason for not being active in the labour market is unemployment or inactivity. Non-employment may impose structural barriers to starting to live with a partner, such as an inability to afford housing. Our first hypothesis (H1) is that *couples in which neither of the partners is employed are less likely to move in together than all other couple configurations*.

*Uncertainty theory.* According to this theoretical framework, the postponement of union formation is driven by uncertainties regarding the individual’s own economic prospects and those of the partner. According to Oppenheimer’s theory of marriage timing (1988), or ‘uncertainty theory’, a dual-earner couple configuration is characterised by negligible uncertainty about whether a certain economic standard and an envisaged lifestyle can be achieved. A lack of uncertainty about having sufficient economic resources may be expected to encourage the transition to co-residence. This leads to hypothesis (H2): *Dual-earner couples are more likely to move in together than other couple configurations.*

*Gendered specialisation and uncertainty reduction.* In a gender-unequal society, the economic prospects of men and women may have different implications for union formation. The male-breadwinner model suggests that men are expected to be the economic provider for the family, and if they fail to do so, they may face social sanctions, feel that their identity is threatened, or report lower life satisfaction (e.g., Kowalewska & Vitali, 2023). According to the specialisation hypothesis (Becker, 1981), if one partner is not employed, the transition to co-residence depends on the other partner’s economic characteristics and will happen when he or she successfully integrates into the labour market (Oppenheimer, 1988). Given that the gendered division of labour implies that men engage in paid labour more than women, the male partner’s employment status might be particularly relevant for the couple’s transition to co-residence, because it functions as a signal of his ability to provide for the couple, and, potentially, for a future family. In line with ‘uncertainty reduction theory’ (Friedman et al., 1994), women with low job-related aspirations or with uncertain economic prospects may invest in family formation as a result of compensatory mechanisms. Empirical research providing support for these theories includes studies based on data from the USA (Sassler et al., 2018), Australia (Bolano & Vignoli, 2021), and Germany (Kurz et al., 2005; Wagner et al., 2019). In a European comparative framework, the importance of men’s resources for union formation depends on the degree of gender equality at the country level (Kalmijn, 2011); accordingly, no differences in the economic characteristics of women and men have been found in the relatively gender-equal context of Finland (Jalovaara, 2012). In the German context in which our study is situated, we expect to find that *male-breadwinner couples have a higher probability of moving in together than jobless couples, but a lower probability than dual-earner couples* (H3). For the opposite couple configuration, which we label ‘female breadwinner’, we expect to observe fewer transitions to co-residence, because moving in with a male partner who is not employed would challenge the gendered specialisation of roles. Still, women’s access to economic resources is relevant because it decreases uncertainty and fosters the establishment of a shared household in the absence of a second income. Indeed, among recent cohorts of young adults, women’s and men’s preferences for partners may have changed, with men increasingly looking for partners with traits associated with labour market success (Oppenheimer, 1988; Van Bavel et al., 2018). Our expectation (H4) is that *female-breadwinner couples have a lower probability of moving in together than dual-earner and male-breadwinner couples, but a higher probability than jobless couples.*

Hypotheses 1–4 are summarised in the table below (Table 1).

**Table 1:** Proposed framework for the association between economic characteristics and the transition to co-residence across configurations of non-cohabiting couples

Hyp. N	Woman’s employment status	Man’s employment status	Association with the transition to co-residence	Reasons
1	Not working	Not working	Negative	Role incompatibility, high structural constraints
2	Employed	Employed	Positive	Uncertainty theory, low structural constraints
3	Not working	Employed	Positive (ref. category)	Gendered specialisation, uncertainty theory, uncertainty reduction theory
4	Employed	Not working	Negative	Gendered specialisation, uncertainty theory

Employment status can obscure important heterogeneities in the economic determinants of cohabitation, especially within the group of employed individuals (see, e.g., Vignoli et al., 2016), as individuals may vary greatly based on their job characteristics. Due to data limitations, we cannot examine these heterogeneities at the couple level, so we focus on them for the main respondent, with analyses stratified by gender. Specifically, we examine two aspects of employment among the subgroup of employed individuals: the nature of their employment contract and their income.

Because moving in together is often the first step towards the institutionalisation of a union, holding a temporary job compared to a permanent job may act as a barrier to union formation: the anticipation of future career uncertainties may favour alternative living arrangements compared to living with a partner. Evidence from the United Kingdom and Australia indicates that having temporary jobs inhibits both young women and men from entering a union (Bolano & Vignoli, 2021; Palumbo et al., 2022). Research from the Netherlands shows that temporarily employed men, but not women, tend to postpone marriage (van Wijk et al., 2021). In Germany, however, temporary employment contracts in the early 2000 s were associated with a probability of transitioning to marriage similar to that of individuals with permanent employment contracts (Kurz et al., 2005). It is likely, then, that the association between temporary jobs and co-residence depends on the contextual labour market specificities faced by young adults. Drawing on uncertainty theories and gendered specialisation, we expect the negative association between having a fixed-term contract and union formation will be weaker for women than for men, leading to the formulation of H5: *Having a temporary employment contract compared to a permanent contract decreases the likelihood of moving in with a partner for both men and women, but the association is weaker for women.*

Finally, having sufficient income has traditionally been seen as a prerequisite for union formation. Like their European peers, young adults in Germany are particularly exposed to the risk of poverty, not only because of their low work intensity, but also due to the lower monetary returns at the early stages of employment careers (Goebel et al., 2015). This financial insecurity may hinder their ability to commit to a co-residential relationship both financially and emotionally. However, little is known about the role played by both partners’ income levels. For younger cohorts, women’s labour income has been confirmed as an important correlate for union formation for the United Kingdom by Palumbo and colleagues (2022); for Finland by Jalovaara (2012); and for the USA with regard to the transition to marriage (Ishizuka, 2018). Yet, the literature discussed above and persistent gender pay gaps in the German context (Schäper et al., 2023) suggest that men’s labour income may play a more decisive role in the decision to moving in together than women’s. Hence, our final hypothesis H6 is as follows: *Higher income is positively associated with the transition to co-residence for both men and women, but the association is stronger for men*. Hypotheses 5–6 are illustrated in Table 2.

**Table 2:** Proposed framework for the association between employment-related characteristics and the transition to co-residence for employed women and men in non-cohabiting couples

Hyp. N	Gender	Employment-related characteristic	Association with the transition to co-residence	Reasons
5	Men	Temporary employment (ref: permanent)	Negative (stronger than for women)	Gendered specialisation, uncertainty theory
	Women	Temporary employment (ref: permanent)	Negative	Gendered specialisation, uncertainty theory, increased labour market participation
6	Men	Income	Positive (stronger than for women)	Gendered specialisation
	Women	Income	Positive	Gendered specialisation, increased labour market participation

## Data and Methods

### Data

The Panel Analysis of Intimate Relationships and Family Dynamics (pairfam) is a survey established in Germany with the aim of studying partnership and family processes. The first wave of data collection, in which around 12,000 individuals living in Germany were interviewed, was conducted in 2008/2009. In the first wave, respondents were from three age groups: 15–17 years, 25–27 years, and 35–37 years, and were thus born in 1991/1993, 1981/1983, and 1971/1973, respectively (Huinink et al., 2011). Those individuals have been interviewed on an annual basis for a maximum of 14 waves; in this paper, we use data from wave 1–13 (collected in 2020/2021).^2^ In wave 11, conducted in 2018/2019, a refreshment sample of individuals born in 2001/2003 was added.

A central feature of pairfam is that it collects detailed information on the partnership and fertility histories of respondents. These data are reorganised into *biopart*, a dataset containing retrospective and prospective monthly-level information about each partnership, including cohabitation and marriage episodes, as well as the characteristics of each previous partner (Brüderl et al., 2022). In addition, a wide variety of data on the main respondents as well as on their current partner are collected at every wave in which they participate in the survey; most relevant for the present study is information on employment status (for both main respondents and their partner), as well as on the nature of the employment contract and income of employed main respondents. Crucially, information on the current partner is available also if the partners live apart. These datasets can be merged into one dataset in long format, which we refer to as relationship-year (one row for each partnership-year, with monthly information on union duration and yearly information on all the other variables).

### Sample Selection

Since we are interested in gender differences within couples and in the interplay between partners’ economic characteristics, different-sex relationships are our unit of analysis. The overall number of relationships reported in the *biopart* dataset, which constitutes the starting point of this analysis, is 37,550. Each respondent can report multiple relationships. Because detailed information on partners preceding the first interview is not provided, and to avoid selection on previous partnerships, we exploit prospective information only, i.e., non-residential relationships that are intact when the main respondent enters the panel or that are formed in the following years in which the main respondent participated in the survey. We hence consider both first and higher-order unions. Relationships that ended or transitioned to cohabitation before the first interview have thus been excluded.

We select relationships that meet the following criteria: they start as non-residential (in 95% of cases the start of the relationship does not coincide with the start of co-residence) and have non-negative^3^ and non-missing information on union duration (74%). We keep those relationships in which the start of cohabitation does not coincide with the start of a marriage (98%), and hence exclude direct marriages, which are extremely rare in our sample. In the remainder of the paper, we use cohabitation and co-residence interchangeably. In addition, the main respondent should be observed at least twice (87%), be above age 10 at the beginning of the relationship, and be above age 17 at the beginning of cohabitation (99%). We also exclude non-cohabiting relationships shorter than three months (N = 721) and longer than 120 months (10 years) (N = 79), as the risk of these partnerships transitioning to co-residence is low. After listwise deletion on missing observations, we obtain a final sample of 14,184 partnership-years from 7165 partnerships formed by 5133 respondents.

In pairfam, information on the nature of the employment contract and income data are only available for main respondents, and not for their partner. For this reason, we model the association between income and type of contract with moving in together on a subsample of relationships reported by either employed women or employed men. These amount to 3108 and 2700 relationship-years, respectively.

### Measurements

Our dependent variable is the hazard of transitioning from an intimate non-residential relationship to non-marital cohabitation; that is, to sharing a dwelling with a partner without being married. Results do not change when we include the 2% relationships that transition to direct marriage (not shown, available from the authors). In pairfam, the presence of an intimate partner is identified through the question: ‘In the following, I’ll ask you about intimate relationships. Do you have a partner in this sense?’ The start of cohabitation is measured by the following questions: ‘Do you live together with (partner) in the same dwelling?’ ‘When did you and (partner) start living together?’.

While the survey has in principle a ‘multi-actor design’ which collects information from main respondents and their partner, the partner’s data suffer from low response rates and selectivity (Schröder et al., 2012). Hence, similar to previous studies (Wagner et al., 2019), we use information provided by the main respondents on their partner’s employment and educational attainment, as these are observable characteristics for which the bias derived from using proxy information should be negligible. The main independent variable is the employment status of both members of the couple: (i) both partners are not employed (because of education or training, inactivity, or unemployment); (ii) the man is employed and the woman is not (‘male breadwinner’); (iii) the woman is employed and the man is not (‘female breadwinner’); and (iv) both partners are employed (‘dual-earner’). In this first set of analyses, the employed category includes self-employed individuals and does not distinguish between full- and part-time employment. In a second set of analyses on the subsample of employed individuals, our main predictors are having a temporary vs. a permanent employment contract and the net labour income from the previous month. Income is recoded into quintiles based on the income distribution of the full sample.

We consider the educational pairing of the couple: (i) homogamy: both low/medium educated partners (including lower secondary education or less than lower secondary and upper secondary/post-secondary); (ii) hypergamy: the man has tertiary education and woman has less than tertiary education; (iii) hypogamy: the woman has tertiary education and the man has less than tertiary education; and (iv) homogamy: both partners are tertiary educated. In this setting, because couples are already formed when first observed and are selected in terms of educational level, it is not possible to study the transition to co-residence as a function of the partners’ education. Rather, we consider the educational pairing of the couple as a control variable to account for differences in the hazard of transitioning to cohabitation. If partnership duration exceeds one wave, given that information on the partner is occasionally missing in the successive waves (2% to 7% of answers), we use the nearest information available in the previous or next wave(s) for gender and education, while assuming it is constant over time, and from the previous wave for employment status only. Information about pregnancy status and age is also available for both partners. Because of the strong correlation between the partners’ ages (Pearson’s correlation = 0.84), the main respondent’s age is used; we include age in both linear and squared form.

The following variables are available for the main respondent only: type of work contract (temporary vs. permanent), monthly net income, number of previous co-residential episodes with other partners, number of children, living in eastern Germany, and age at the start of the relationship. The number of children refers to children currently co-residing with the main respondent, with no distinction between biological, adopted, or stepchildren, and is recoded into a categorical variable with three categories: childless, one child, and two or more children. The number of previous cohabitation (or marriage) episodes is measured on the previous wave to avoid collinearity with the outcome. Because women’s employment and relationship patterns continue to differ between eastern and western Germany (Fulda, 2016), we control for the main respondent’s region of residence (east vs. west). All variables are time-varying at the yearly level, roughly corresponding to the between-wave interval of the panel.

### Methods

To study the transition to cohabitation we use event-history analysis, which allows us to jointly model the likelihood of the event happening and the pace of the process. Couples start to be at risk of moving in together (i.e., cohabiting) when they are formed; that is, at the beginning of their relationship, as reported by the main respondent. Time to the event of co-residence is measured in months and each couple can move in together only once (single-failure data). The hazard of moving in together is hence a function of union duration. Following related studies (van der Wiel et al., 2020; Wagner et al., 2019), we consider an episode to be censored if the relationship continues as non-cohabiting in the last wave when it is observed, or if the couple separates and therefore does not proceed to cohabitation. We treat separation as a cause for censoring because we are not focused on the determinants of separation of non-residential couples, which are likely to differ from the determinants of living together (Ciritel, 2022).

In a first step, we describe couples’ transitions to cohabitation by their combined economic characteristics using Kaplan–Meier survival analysis, which does not account for confounding variables. In a second step, we use Cox proportional hazards regression (Cox, 1972) with standard errors clustered by individual to correct for the fact that multiple relationships can be reported by the same respondent. The main feature of Cox models is that the baseline hazard is left unspecified and that the covariates are multiplicatively related to the hazard; the proportionality of the hazard functions is assumed to be constant over time (Cox, 1972). We estimate three regression models on our data in relationship-year format: Model 1, our baseline model, is run on the overall sample and allows us to jointly consider the employment status of both partners; Model 2 is restricted to the relationships reported by employed individuals and is performed separately on women and men. We also run a model on all employed individuals in Model 3, including interactions terms to check for gender differences. As robustness checks (presented in the Appendix), we consider alternative model specifications, test the assumption of proportional hazards for the main covariates of interest, and restrict the analyses to the relationships started after age 20 to check whether our results are driven by the relationships reported by younger respondents. We also run our main models with competing risks for cohabitation vs. separation to ensure that our predictors are related to the main event of interest (cohabitation) and not to its competing event (separation).

## Results

### Descriptive Results

Table 3 describes the composition of our analytical sample of relationships across all the years in which one relationship was reported. It shows that 60% of non-residential relationships are reported by main respondents born in 1991–1993, with a mean age of 23 at the start of the relationship and a mean age of 25 throughout the observation window; 55% of respondents have never cohabited and 90% of them are childless. However, the proportion of respondents in their late thirties is not negligible: 10% of them are aged 37 or older. The sample is slightly skewed towards female respondents (55%). In terms of employment status, the composition of the couples is quite diverse: jobless couples are most prevalent (37%), primarily because of participation in education or training at younger ages, followed by dual earners (31%). Among couples with only one employed partner, male breadwinners (21%) are more common than female breadwinners (11%). These results reflect labour market participation patterns of German couples, among whom gendered specialisation is uncommon before the transition to parenthood. In terms of educational attainment, couples in which both partners have low or medium education are the largest group (58%), reflecting the young age and the prevalence of ongoing enrolment of our sample. Among employed respondents, 25% of men and 26% of women have a temporary job. The median net monthly income reported is €1,585 for employed men and €1,230 for employed women, reflecting the average gender pay gap in Germany.

**Table 3:** Descriptive statistics (unweighted)

	Summary statistics
Birth cohort of main respondent	
1971–1973	1347 (9.5%)
1981–83	2880 (20.3%)
1991–93	8521 (60.1%)
2001–2003	1436 (10.1%)
Sex of main respondent	
Male	6456 (45.5%)
Female	7728 (54.5%)
No. of waves in which main respondent participated	9.148 (3.977)
Age of main respondent at the beginning of the relationship	23.041 (7.386)
Age of main respondent (time-varying)	24.945 (7.513)
Number of previous cohabitations of main respondent	0.604 (0.789)
Child(ren) co-resident with main respondent	
Childless	12,803 (90.3%)
One child	803 (5.7%)
Two or more children	578 (4.1%)
Main respondent currently living in eastern Germany	
No	11,531 (81.3%)
Yes	2653 (18.7%)
Couples'combined economic characteristics	
Both not working	5307 (37.4%)
M employed, W not working	3008 (21.2%)
M not working, W employed	1515 (10.7%)
Both employed	4354 (30.7%)
Couples'educational pairing	
Both low/medium educated	8167 (57.6%)
M tertiary educated, W less than tertiary educated	1904 (13.4%)
W tertiary educated, M less than tertiary educated	1695 (12.0%)
Both tertiary educated	2418 (17.0%)
Couple is expecting a child	
No	13,961 (98.4%)
Yes	223 (1.6%)
*N (Relationship-years)*	14,184

**Fig. 1:**
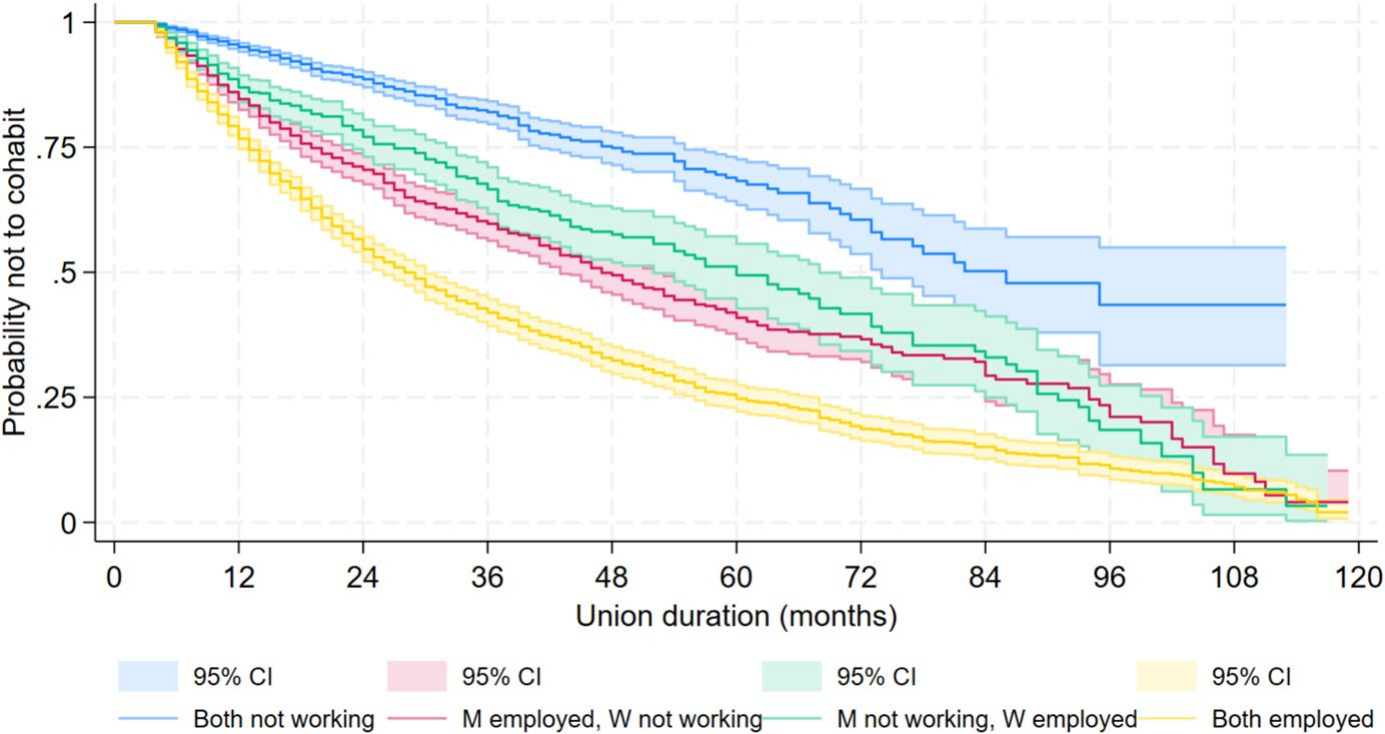
Probability of surviving to cohabitation (i.e., to continue living apart), by non-residential partners’ combined economic characteristics (Kaplan–Meier estimates) *Notes:* ‘Not working’ refers to being a student, unemployed, or inactive. ‘Employed’ refers to having an employment contract, with no distinction between employment and self-employment, full-time and part-time employment

Figure 1 shows Kaplan–Meier survival curves to co-residence for couples with different employment configurations as a function of union duration, expressed in months since the start of the relationship. The slope of the survival curve is flat for jobless couples, indicating a lower rate of transitioning to co-residence at any duration of the union. The slope of the survival curve is steepest for couples with two employed partners, who tend to move in together earlier than the other couple types, particularly at shorter union durations. If only the male partner is employed, the transition to cohabitation is slower compared to dual earners, but it is faster compared to jobless couples at any union duration. Female-breadwinner couples have a slightly lower probability of transitioning to cohabitation than male-breadwinner couples, but the shapes of the survival curves look remarkably similar, particularly at longer union durations.

### Multivariate Results

**Fig. 2:**
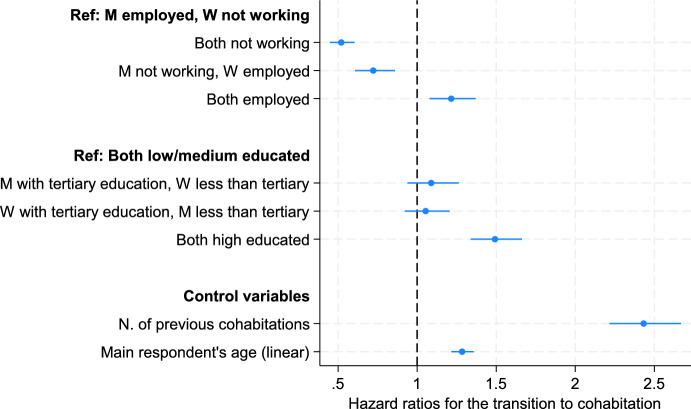
Hazard of moving in together by non-residential partners’ combined characteristics. Coefficients from Cox regression, Model 1 (Table 4) *Notes:* N = 14,184 relationship-years. The model additionally controls for: main respondent or partner expecting a child, n. of children living with respondent, respondent living in eastern Germany, respondent’s age (squared)

The Cox regression model accounts for the compositional heterogeneity of non-residential couples by including a set of control variables. Results (reported in Table 4, main coefficients shown in Fig. 2) confirm the patterns revealed by Kaplan–Meier estimates. Jobless couples display the lowest propensity to transition to cohabitation: their hazards are 48% lower than those of male-breadwinner couples (i.e., the reference category). Dual-earner couples are 22% more likely to transition to cohabitation than male-breadwinner couples. Contrary to the finding of Kaplan–Meier estimates that male- and female-breadwinner couples have a similar hazard of transitioning to co-residence, when the set of control variables is included, female-breadwinner couples are 28% less likely to transition to cohabitation than male-breadwinner couples. This indicates that in single-breadwinner couples, the gender of the employed partner is differently associated with moving in together. Switching the reference category reveals that all groups differ significantly from each other (results not shown).

**Table 4:** Cox proportional hazard model on the transition to cohabitation. Model 1, hazard ratios

	Hazard ratios
Couples’ economic characteristics	
(ref: M employed, W not working)	
*Both not working*	0.520***
	(0.0400)
*M not working, W employed*	0.723***
	(0.0640)
*Both employed*	1.216**
	(0.0741)
Couples’ educational attainment (ref: Both low/medium educated)	
*M tertiary educated, W less than tertiary educated*	1.089
	(0.0826)
*W tertiary educated, M less than tertiary educated*	1.054
	(0.0725)
*Both tertiary educated*	1.492***
	(0.0825)
Couple expecting a child	1.887***
	(0.252)
Number of child(ren) living with main respondent (ref: childless)	
*One child*	1.056
	(0.130)
*Two or more children*	0.724*
	(0.113)
Number of main respondent’s previous cohabitations	2.432***
	(0.115)
Main respondent living in eastern Germany	1.028
	(0.0601)
Main respondent’s age (linear)	1.284***
	(0.0359)
Main respondent’s age (squared)	0.995***
	(0.000468)
*N (relationship-years)*	14,184

Exponentiated coefficients; robust standard errors in parentheses

* *p* < 0.05, ** *p* < 0.01, *** *p* < 0.001

Findings regarding the control variables are in line with prior research on non-economic determinants of union formation. Homogamous couples with high levels of education exhibit a 49% higher hazard of transitioning from living apart to living together as a cohabiting couple than homogamous couples with low or medium education (the reference group). Heterogamous couples do not differ from the reference group, or from homogamous highly educated couples (results not shown). Both older respondents and those with previous cohabitation/marriage experience show an increased hazard of cohabiting. While expecting a child considerably increases the hazard of transitioning to cohabitation (by 88%), the presence of two or more child(ren) residing with the main respondent (probably from a previous union) slightly deters moving in together compared to being childless. The hazard of cohabiting does not differ between respondents living in eastern and western Germany, signalling that distinct partnership trajectories between East and West only emerge after the transition to cohabitation (Fulda, 2016; Hiekel et al., 2015).

**Fig. 3:**
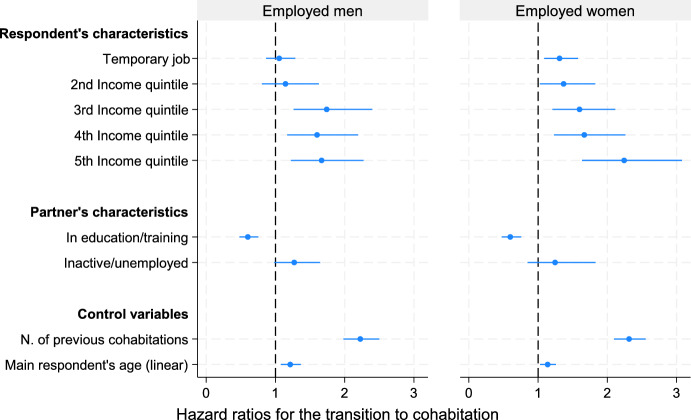
Hazard of moving in together among employed respondents in a non-residential relation, by gender. Coefficients from Cox regression, Model 2 (Table 5) *Notes:* N (men) = 2700 relationship-years, N (women) = 3108. The model additionally controls for: main respondent or partner expecting a child, n. of children living with respondent, respondent living in eastern Germany, respondent’s age (squared)

Next, to address heterogeneity among employed respondents, we estimate two Cox regression models, separately for men and women, on the subset of couples in which the main respondent is employed (results presented in Table 5, main coefficients shown in Fig. 3). In this model, we introduce the nature of the employment contract (permanent vs. temporary) and income levels in quintiles, while controlling for the employment status and the educational attainment of both partners. Compared to non-residential couples in which women have a permanent employment contract, those in which women have a temporary contract exhibit an increased hazard of moving in together (by 35.5%). Results for men are in a similar direction but are not statistically significant. A higher income is positively associated with moving in together: both women and men in the fifth income quintile exhibit a 66% higher hazard of starting a non-marital cohabitation compared to the reference category (first quintile).

**Table 5:** Cox proportional hazard model on the transition to cohabitation, employed men and women. Model 2, hazard ratios

	Hazard ratios—Men	Hazard ratios—Women
Temporary job (ref: permanent)	1.054	1.355**
	(0.107)	(0.127)
Partner’s employment status (ref: employed)		
*In education/training*	0.601***	0.670***
	(0.0688)	(0.0782)
*Inactive/unemployed*	1.271	1.254
	(0.168)	(0.239)
Main respondent’s educational attainment (ref: upper secondary/post-secondary)		
*Lower secondary or less*	1.047	0.884
	(0.190)	(0.164)
*Tertiary*	1.228*	1.010
	(0.111)	(0.0921)
Partner’s educational attainment (ref: upper secondary/post-secondary)		
*Lower secondary or less*	1.202	1.074
	(0.176)	(0.222)
*Tertiary*	1.039	1.015
	(0.0961)	(0.0878)
Income quintiles (net from prev. month), ref: first quintile		
*Second quintile*	1.145	1.363*
	(0.206)	(0.187)
*Third quintile*	1.740***	1.576***
	(0.285)	(0.211)
*Fourth quintile*	1.601**	1.504**
	(0.257)	(0.221)
*Fifth quintile*	1.666**	1.659**
	(0.264)	(0.255)
Couple expecting a child	1.689*	1.484
	(0.357)	(0.414)
Number of child(ren) living with main respondent (ref: childless)		
*One child*	2.405***	0.302***
	(0.435)	(0.0500)
*Two or more children*	1.715*	0.343***
	(0.408)	(0.0775)
Number of main respondent’s previous cohabitations	2.225***	1.964***
	(0.132)	(0.0967)
Main respondent living in eastern Germany	0.969	1.098
	(0.0986)	(0.111)
Main respondent’s age (linear)	1.213**	1.138*
	(0.0738)	(0.0586)
Main respondent’s age (squared)	0.996***	0.9969***
	(0.000969)	(0.000811)
*N (relationship-years)*	2700	3108

Exponentiated coefficients; robust standard errors in parentheses

*p < 0.05, ** *p* < 0.01, *** *p* < 0.001

In order to compare women and men and to test our hypotheses regarding gender differences, the same model is estimated jointly on the sample of employed women and men (Table A1, Appendix), adding an interaction between gender and temporary contracts (Model 3a), and between gender and income (Model 3b). The coefficient of the interaction between gender and temporary contracts is statistically significant (H.R. = 1.308, *p* = 0.038), showing that couples in which female respondents have temporary contracts are slightly more likely (by 4%) to transition to cohabitation than couples where male respondents have temporary contracts. No differences emerge between women and men in the association between income quintiles and the hazard of cohabiting.

Concerning the model estimated on women and men separately, results regarding the control variables (age, previous cohabitation/marriage, eastern vs. western Germany) are in line with the model estimated on the full sample of employed and non-employed individuals. Results do deviate from the full sample for educational attainment, employment status, and presence of children. Tertiary-educated and employed men have a slightly higher hazard of transitioning to cohabitation than men with lower educational attainment, but this is not the case for tertiary-educated and employed women. Moreover, having a tertiary-educated partner (either male or female) is not associated with the hazard of cohabiting. The hazard of cohabiting is substantially lower (by around 40%) if the female or male partner is in education or training rather than being employed, while having an unemployed or inactive partner does not lower the chances of cohabiting among this subsample. The presence of children is negatively associated with the transition to cohabitation for women, while the association is positive for men.

In order to rule out possible confounding effects between the two considered employment-related characteristics (van Wijk et al., 2021), we also estimate separate Cox regression models including either contract type or income. The association between the type of employment contract and the hazard of cohabiting without controlling for income is consistent with the full model (not shown for brevity): temporary employment is not associated with the transition to cohabitation for men (H.R. = 0.963, *p* = 0.707), while it is positively associated for women (H.R. = 1.231, *p* = 0.024).

### Robustness Checks

Our baseline model addressing the association between couples’ configurations and the transition to cohabitation (Model 1) has been estimated with an additional control for age differences between the partners. The coefficient of the age difference is not statistically significant (not reported for brevity), signalling that the age of the partner does not confound the relationship we are interested in. To rule out the possibility that our results are driven by the relationship behaviour of younger respondents, we also estimate the baseline model on those relationships that started when the main respondent was aged 20 or older, and was thus above the median age at the start of the relationship in our sample. After this restriction is applied, the sample size drops to 7546 relationship-years. Results shown in Table A2, Appendix remain consistent with those obtained for the full sample for all the independent and control variables, although the effect of having children disappears, and age (in its linear specification) is no longer significant.

Furthermore, we test whether the assumption of proportional hazards is violated for the main covariates of interest, meaning that the implied association between such variables and the outcome is not constant over time, but rather depends on union duration. On the basis of log–log plots and Schoenfeld residuals it can be concluded that the assumption is not violated for couples’ economic characteristics or for educational attainment. Violations of the proportional hazard assumption are observed among the control variables related to pregnancy and the number of co-resident children. While these results could hint at a composite role played by the child’s age and union duration, they do not constitute the focus of the paper. Therefore, we do not conduct stratified analyses based on these variables.

To ensure that our covariates of interest are not associated with couples’ risk of separation, an event that we are treating as censored, we furthermore run Model 1 with competing risks for cohabitation vs. separation (Table A3, Appendix). Results show that the couples’ composition in terms of employment status is associated with the transition to cohabitation, but not with separation. Educational attainment is instead related to separation: couples in which both partners are low/medium educated have higher separation hazards than others. When we replicate Model 2 with competing risks, neither temporary employment nor income is associated with separation (Table A4, Appendix).

## Discussion

Over the last few decades, partnering behaviours, as well as patterns of educational and labour market participation, have undergone profound transformations in Europe. These changes have been particularly pronounced among young adults, who face increasing difficulties in securing stable employment, while simultaneously navigating romantic relationships. The existing literature has not fully captured the complexity of the transition to co-residential unions and its connection to economic uncertainty among recent cohorts. In this paper, we used unique dyadic data on different-sex, non-residential couples from the German Family panel (pairfam), to examine couples’ transitions to co-residence using event-history models. We build on previous literature considering the role of multiple employment-related characteristics (i.e., employment status, type of employment contract, income) of partnered women and men in non-residential unions.

The likelihood of transitioning to cohabitation varies considerably between different couple configurations based on employment status, in line with hypotheses on role incompatibility and uncertainty (Hypotheses 1 and 2, Table 1). Dual-earner couples in Germany have the lowest levels of economic insecurity and the lowest risk of poverty (Goebel et al., 2015) and are generally better able to overcome the structural barriers associated with access to a rented dwelling. Unsurprisingly, they tend to transition to cohabitation faster than other couple constellations. In accordance with Oppenheimer’s theory of marriage timing (1988), but also in line with the notion of role incompatibility (Blossfeld & Huinink, 1991), the completion of education is a prerequisite for moving in together: analyses of employed individuals show that having a partner still enrolled in education substantially reduces the likelihood of moving in together compared to unemployment or inactivity. The finding that jobless couples exhibit the lowest transition probabilities suggests that these couples, predominantly composed by individuals currently enrolled in education, postpone moving in together until they reach a more stable life course stage (Régnier-Loilier et al., 2009). The inclusion of both partners’ economic characteristics allowed us to address gendered associations between employment and union formation. In line with Hypotheses 3 and 4 (Table 1) on gendered specialisation, the fact that the man is employed (dual-earner or male-breadwinner couples) represents a precondition for moving in together, whereas couples in which the man is not employed (jobless or female-breadwinner couples) have a reduced likelihood of moving in together. Hence, consistent with previous research (Kurz et al., 2005; Wagner et al., 2019), our findings indicate that the male-breadwinner arrangement continues to predict the transition to cohabitation, even among younger cohorts. However, dual earning by far represent the preferred couple’s employment arrangement for moving in together, suggesting a growing preference for economic homogamy and the need of two salaries for establishing a co-residential household with a partner.

When analysing heterogeneity in the likelihood of cohabitation among employed respondents, we found that in Germany, unlike in other countries (Bolano & Vignoli, 2021; Palumbo et al., 2022), having a temporary employment contract increases the likelihood of moving in together for women, but not for men. This finding contradicts Hypothesis 5. It suggests that having a temporary contract does not signal economic precariousness and does not discourage individuals from committing to cohabitation. This result can be interpreted in light of the observation that, in Germany, temporary employment is common at the beginning of the work career, including among high-skilled individuals (Kurz et al., 2005). Among younger cohorts, any type of employment, rather than secure employment, is a prerequisite for the transition to non-marital cohabitation. The observed gender difference in the role of temporary employment is small, but can be explained by the uncertainty reduction theory (Friedman et al., 1994): women with a more uncertain contract type might be more willing to invest in union formation to safeguard their economic status through their partner, in line with gendered perceptions about economic dependence. The positive association found between income and cohabitation among both employed women and employed men confirms previous findings (Jalovaara, 2012; Palumbo et al., 2022) and highlights that also in Germany the transition to co-residence is socially stratified. While this result is in line with the expected positive association between income and the transition to cohabitation, it dismisses the hypothesis of a gendered role of labour income illustrated in Hypothesis 6. Earning a higher salary appears to be a prerequisite for moving in together, for women as well as for men.

We acknowledge several limitations. First, since information on contract type and income was only available for the main respondents, we were unable to study these factors as relative employment characteristics within couples. This limitation was addressed by stratifying our analyses by gender, which allowed us to evaluate how employment-related characteristics are differently associated with the transition to a co-residential union among women and men. Second, although event-history models recognise antecedent variables, they do not allow us to study whether within-couple transitions in the explanatory variables (e.g., from both partners being employed to both being unemployed) are related to the transition to cohabitation. Instead, they only assess whether being in a particular state (e.g., both employed) is associated with the outcome of interest. Given that monthly-level information about partners’ employment status is not available in pairfam, couple configurations at the yearly level represent the maximum level of granularity available in the data, and allowed us to grasp how partnerships evolve according to the partners’ economic characteristics. Third, the role of contextual factors that may affect the decision to cohabit such as housing prices cannot be examined with pairfam because of limited information on respondents’ and their partners’ place of residence and living arrangements.

Our findings have important implications. First, if young people struggle to enter the labour market, they may delay cohabitation and the subsequent steps in relationship progression (Esteve et al., 2020). Unemployment or low earnings may affect certain segments of the population more than others; in Germany, young men with low educational attainment are particularly vulnerable, given their decreased chances of entering a co-residential union (Corti & Scherer, 2021). Although this study could not assess whether there is a general preference for postponing union formation until labour market entry—prioritising single living or co-residence with parents—our results suggest structural barriers to establishing a joint household, reinforced by normative beliefs about the role of the man as the family’s economic provider. Second, as cohabitation has become normative as a precursor to marriage, it is now associated with more substantial economic resources, which were once considered a requirement exclusively for marriage (Jalovaara, 2012). Given the changing circumstances surrounding union formation among young adults, it is evident that previous theories attempting to explain the transition to cohabitation as an alternative to marriage need to be revisited (Manning, 2020). Therefore, the study of partnership processes must consider non-residential relationships and the steps that precede cohabitation (Bergström & Moulin, 2022; Manning, 2020; Sassler, 2010). Being single, living apart together, cohabiting, and getting married represent distinct states of partnership progression, each of which can be repeated over the life course, and whose meaning and determinants can vary substantially over time and across different contexts. New research avenues open up as data collecting information on the characteristics of respondents’ non-residential partners become available, as in the case of pairfam. To advance our theoretical understanding of complex partnership trajectories and to foster comparative research, longitudinal data from other countries are needed, including detailed information about both partners in non-residential relationships. Data on unpartnered individuals are also essential to understand contemporary trends of (permanent) singlehood and the inequalities therein as a potential driver of postponing or foregoing family formation.

## Supplementary Information

Below is the link to the electronic supplementary material.

Supplementary file1 (DOCX 34 KB)

## Data Availability

The datasets analysed during the current study are freely available upon signature of a data use agreement and therefore cannot be shared by authors. More information about data access can be found here: https://www.pairfam.de/en/data/data-access/.

## Footnotes

1 This broad definition follows the review study by Giraud (2023) as well as empirical research (Castro-Martín et al., 2008; Régnier-Loilier et al., 2009; Wagner et al., 2019).

2 In this paper, we use data from the German Family Panel (pairfam), release 13.0 (Brüderl et al., 2022), coordinated by Josef Brüderl, Sonja Drobnič, Karsten Hank, Johannes Huinink, Bernhard Nauck, Franz J. Neyer, and Sabine Walper. The study was funded from 2004 to 2022 as a priority program and a long-term project by the German Research Foundation (DFG). A detailed description of the study is provided by Huinink et al. (2011).

3 A negative union duration is recorded when the start of cohabitation precedes the start of the relationship.
