# Supplementary material for: Non-cohabiting Partners’ Economic Characteristics and the Transition to Living Together in Germany: A Couple-Level Perspective
Source: Eur J Popul. 2025 Oct 8;41(1):25. doi: 10.1007/s10680-025-09740-y (PMC12508365; doi:10.1007/s10680-025-09740-y)
Supplement: Supplementary file 1 — Supplementary file1 (DOCX 34 KB) [file 10680_2025_9740_MOESM1_ESM.docx]

**Appendix**

Table A1: Cox proportional hazard model on the transition to cohabitation, employed men and women, combined. Model 3, hazard ratios

|  | Model without interactions, Hazard ratios | Model 3a,  Hazard ratios | Model 3b,  Hazard ratios |
| --- | --- | --- | --- |
| Women | 0.851^**^ | 0.799^***^ | 0.858 |
|  | (0.0507) | (0.0543) | (0.148) |
|  |  |  |  |
| Temporary job (ref: permanent) | 1.149^*^ | 0.991 | 1.151^*^ |
|  | (0.0788) | (0.0957) | (0.0793) |
|  |  |  |  |
| Partner’s employment status (ref: employed) |  |  |  |
| *In education/training* | 0.591^***^ | 0.596^***^ | 0.590^***^ |
|  | (0.0487) | (0.0490) | (0.0487) |
| *Inactive/unemployed* | 1.510^***^ | 1.510^***^ | 1.509^***^ |
|  | (0.153) | (0.153) | (0.153) |
|  |  |  |  |
| Main respondent’s educational attainment (ref: upper secondary/post-secondary) |  |  |  |
| *Lower secondary or less* | 0.865 | 0.850 | 0.857 |
|  | (0.122) | (0.122) | (0.122) |
| *Tertiary* | 1.147^*^ | 1.141^*^ | 1.141^*^ |
|  | (0.0738) | (0.0736) | (0.0736) |
|  |  |  |  |
| Partner’s educational attainment (ref: upper secondary/post-secondary) |  |  |  |
| *Lower secondary or less* | 1.212 | 1.220 | 1.208 |
|  | (0.146) | (0.147) | (0.144) |
| *Tertiary* | 1.024 | 1.026 | 1.026 |
|  | (0.0644) | (0.0646) | (0.0647) |
|  |  |  |  |
| Income quintiles (net from prev. month), ref: First quintile |  |  |  |
| *Second quintile* | 1.261^*^ | 1.264^*^ | 1.141 |
|  | (0.145) | (0.145) | (0.200) |
| *Third quintile* | 1.722^***^ | 1.725^***^ | 1.846^***^ |
|  | (0.185) | (0.185) | (0.290) |
| *Fourth quintile* | 1.707^***^ | 1.708^***^ | 1.747^***^ |
|  | (0.190) | (0.191) | (0.272) |
| *Fifth quintile* | 2.003^***^ | 1.988^***^ | 1.946^***^ |
|  | (0.225) | (0.224) | (0.298) |
|  |  |  |  |
| Main respondent or partner expecting a child | 1.540^**^ | 1.567^**^ | 1.553^**^ |
|  | (0.243) | (0.245) | (0.247) |
|  |  |  |  |
| Number of child(ren) living with main respondent (ref: childless) |  |  |  |
| *One child* | 0.940 | 0.933 | 0.940 |
|  | (0.124) | (0.123) | (0.124) |
| *Two or more children* | 0.868 | 0.873 | 0.854 |
|  | (0.149) | (0.151) | (0.148) |
|  |  |  |  |
| Number of main respondent’s previous cohabitations | 2.227^***^ | 2.226^***^ | 2.232^***^ |
|  | (0.0844) | (0.0846) | (0.0845) |
|  |  |  |  |
| Main respondent living in eastern Germany | 0.997 | 0.999 | 0.998 |
|  | (0.0734) | (0.0731) | (0.0733) |
|  |  |  |  |
| Main respondent’s age (linear) | 1.140^***^ | 1.143^***^ | 1.142^***^ |
|  | (0.0441) | (0.0445) | (0.0444) |
|  |  |  |  |
| Main respondent’s age (squared) | 0.997^***^ | 0.997^***^ | 0.997^***^ |
|  | (0.000609) | (0.000613) | (0.000612) |
|  |  |  |  |
| Woman # temporary job |  | 1.308^*^ |  |
|  |  | (0.170) |  |
|  |  |  |  |
| Woman # 2^nd^ income quintile |  |  | 1.157 |
|  |  |  | (0.261) |
|  |  |  |  |
| Woman # 3^rd^ income quintile |  |  | 0.877 |
|  |  |  | (0.178) |
|  |  |  |  |
| Woman # 4^th^ income quintile |  |  | 0.946 |
|  |  |  | (0.198) |
|  |  |  |  |
| Woman # 5^th^ income quintile |  |  | 1.086 |
|  |  |  | (0.222) |
| *N (relationship-years)* | 5,808 | 5,808 | 5,808 |

Exponentiated coefficients; robust standard errors in parentheses

^*^ *p* < 0.05, ^**^ *p* < 0.01, ^***^ *p* < 0.001

Table A2: Cox proportional hazard model on the transition to cohabitation, main respondent aged 20+ at the start of the relationship. Model 1, hazard ratios

|  | Hazard ratios |
| --- | --- |
| Couples’ economic characteristics |  |
| (ref: M employed, W not working) |  |
| *Both not working* | 0.605^***^ |
|  | (0.0634) |
| *M not working, W employed* | 0.714^***^ |
|  | (0.0731) |
| *Both employed* | 1.269^***^ |
|  | (0.0846) |
|  |  |
| Couples’ educational attainment (ref: Both low/medium educated) |  |
| *M tertiary educated, W less than tertiary educated* | 1.080 |
|  | (0.0841) |
| *W tertiary educated, M less than tertiary educated* | 1.084 |
|  | (0.0789) |
| *Both tertiary educated* | 1.405^***^ |
|  | (0.0848) |
|  |  |
| Main respondent or partner expecting a child | 1.807^***^ |
|  | (0.252) |
|  |  |
| Number of child(ren) living with main respondent (ref: childless) |  |
| *One child* | 0.994 |
|  | (0.113) |
| *Two or more children* | 0.760 |
|  | (0.109) |
|  |  |
| Number of main respondent’s previous cohabitations | 2.116^***^ |
|  | (0.0867) |
|  |  |
| Main respondent living in eastern Germany | 0.956 |
|  | (0.0599) |
|  |  |
| Main respondent’s age (linear) | 1.040 |
|  | (0.0443) |
|  |  |
| Main respondent’s age (squared) | 0.998^*^ |
|  | (0.000651) |
| *N (relationship-years)* | 7,546 |

Exponentiated coefficients; robust standard errors in parentheses

^*^ *p* < 0.05, ^**^ *p* < 0.01, ^***^ *p* < 0.001

Table A3: Cohabitation and separation analyses from Cox proportional hazards model. Model 1, hazard ratios

|  | Hazard ratios for | Hazard ratios for |
| --- | --- | --- |
|  | cohabitation | separation |
| Couples’ economic characteristics |  |  |
| (ref: M employed, W not working) |  |  |
| *Both not working* | 0.511^***^ | 1.072 |
|  | (0.0388) | (0.0513) |
| *M not working, W employed* | 0.700^***^ | 1.115 |
|  | (0.0615) | (0.0698) |
| *Both employed* | 1.192^**^ | 0.902 |
|  | (0.0718) | (0.0571) |
|  |  |  |
| Couples’ educational attainment (ref: Both low/medium educated) |  |  |
| *M tertiary educated, W less than tertiary educated* | 1.095 | 0.884^*^ |
|  | (0.0799) | (0.0509) |
| *W tertiary educated, M less than tertiary educated* | 1.041 | 0.806^**^ |
|  | (0.0707) | (0.0532) |
| *Both tertiary educated* | 1.471^***^ | 0.777^***^ |
|  | (0.0809) | (0.0488) |
|  |  |  |
| Main respondent or partner expecting a child | 1.802^***^ | 0.478^**^ |
|  | (0.243) | (0.119) |
| Number of child(ren) living with main respondent (ref: childless) |  |  |
| *One child* | 1.060 | 1.700^***^ |
|  | (0.129) | (0.183) |
| *Two or more children* | 0.758 | 1.017 |
|  | (0.113) | (0.173) |
|  |  |  |
| Number of main respondent’s previous cohabitations | 2.394^***^ | 0.399^***^ |
|  | (0.109) | (0.0250) |
|  |  |  |
| Main respondent living in eastern Germany | 1.043 | 1.120^*^ |
|  | (0.0600) | (0.0514) |
|  |  |  |
| Main respondent’s age (linear) | 1.286^***^ | 0.880^***^ |
|  | (0.0345) | (0.0185) |
|  |  |  |
| Main respondent’s age (squared) | 0.995^***^ | 1.002^***^ |
|  | (0.000449) | (0.000353) |
| *N (relationship-years)* | 14,526 | 14,526 |

Exponentiated coefficients; robust standard errors in parentheses

^*^ *p* < 0.05, ^**^ *p* < 0.01, ^***^ *p* < 0.001

Table A4: Cohabitation and separation analyses from Cox proportional hazards model, employed men and women. Model 2, hazard ratios

|  | Model 2 – Men | | Model 2 – Women | |
| --- | --- | --- | --- | --- |
|  | Hazard ratios for  cohabitation | Hazard ratios for  separation | Hazard ratios for  cohabitation | Hazard ratios for  separation |
| Temporary job (ref: permanent) | 1.095 | 1.043 | 1.304^**^ | 1.158 |
|  | (0.109) | (0.113) | (0.121) | (0.111) |
|  |  |  |  |  |
| Partner’s employment status (ref: employed) |  |  |  |  |
| *In education/training* | 0.602^***^ | 1.228 | 0.593^***^ | 1.189 |
|  | (0.0681) | (0.143) | (0.0702) | (0.129) |
| *Inactive/unemployed* | 1.254 | 1.044 | 1.223 | 1.024 |
|  | (0.161) | (0.224) | (0.234) | (0.197) |
|  |  |  |  |  |
| Main respondent’s educational attainment (ref: upper secondary/post-secondary) |  |  |  |  |
| *Lower secondary or less* | 1.061 | 1.311 | 0.790 | 0.933 |
|  | (0.191) | (0.204) | (0.150) | (0.182) |
| *Tertiary* | 1.218^*^ | 1.021 | 0.939 | 0.918 |
|  | (0.108) | (0.124) | (0.0837) | (0.113) |
|  |  |  |  |  |
| Partner’s educational attainment (ref: upper secondary/post-secondary) |  |  |  |  |
| *Lower secondary or less* | 1.170 | 1.003 | 1.209 | 1.231 |
|  | (0.169) | (0.164) | (0.245) | (0.193) |
| *Tertiary* | 1.043 | 0.815 | 1.037 | 0.887 |
|  | (0.0952) | (0.103) | (0.0857) | (0.0897) |
|  |  |  |  |  |
| Income quintiles (net from prev. month), ref: First quintile |  |  |  |  |
| *Second quintile* | 1.145 | 0.916 | 1.366^*^ | 1.195 |
|  | (0.203) | (0.133) | (0.195) | (0.158) |
| *Third quintile* | 1.780^***^ | 0.845 | 1.611^***^ | 1.157 |
|  | (0.287) | (0.123) | (0.224) | (0.176) |
| *Fourth quintile* | 1.574^**^ | 0.750 | 1.715^***^ | 1.047 |
|  | (0.251) | (0.130) | (0.261) | (0.189) |
| *Fifth quintile* | 1.712^***^ | 0.688^*^ | 2.236^***^ | 0.953 |
|  | (0.269) | (0.116) | (0.354) | (0.216) |
|  |  |  |  |  |
| Main respondent or partner expecting a child | 1.374 | 0.371 | 1.456 | 0.245 |
|  | (0.329) | (0.226) | (0.383) | (0.236) |
|  |  |  |  |  |
| Number of child(ren) living with main respondent (ref: childless) |  |  |  |  |
| *One child* | 2.473^***^ | 1.410 | 0.509^***^ | 2.210^***^ |
|  | (0.433) | (0.494) | (0.0885) | (0.423) |
| *Two or more children* | 1.709^*^ | 0.909 | 0.602^*^ | 1.015 |
|  | (0.388) | (0.343) | (0.127) | (0.284) |
|  |  |  |  |  |
| Number of main respondent’s previous cohabitations | 2.189^***^ | 0.470^***^ | 2.258^***^ | 0.452^***^ |
|  | (0.124) | (0.0771) | (0.108) | (0.0456) |
|  |  |  |  |  |
| Main respondent living in eastern Germany | 0.999 | 1.147 | 1.022 | 1.048 |
|  | (0.100) | (0.152) | (0.102) | (0.141) |
|  |  |  |  |  |
| Main respondent’s age (linear) | 1.239^***^ | 0.827^***^ | 1.126^*^ | 0.996 |
|  | (0.0746) | (0.0468) | (0.0560) | (0.0544) |
|  |  |  |  |  |
| Main respondent’s age (squared) | 0.996^***^ | 1.003^***^ | 0.997^***^ | 1.000 |
|  | (0.000956) | (0.000893) | (0.000782) | (0.000843) |
| *N (relationship-years)* | 2,764 | 2,764 | 3,171 | 3,171 |

Exponentiated coefficients; robust standard errors in parentheses

^*^ *p* < 0.05, ^**^ *p* < 0.01, ^***^ *p* < 0.001
